# Sparse somatosensory coding: towards explaining and predicting the response properties of rodent afferent pathway neurons

**DOI:** 10.1186/1471-2202-14-S1-P127

**Published:** 2013-07-08

**Authors:** Mathew H Evans

**Affiliations:** 1 Psychology Department, University of Sheffield, UK

## 

**Figure 1:**
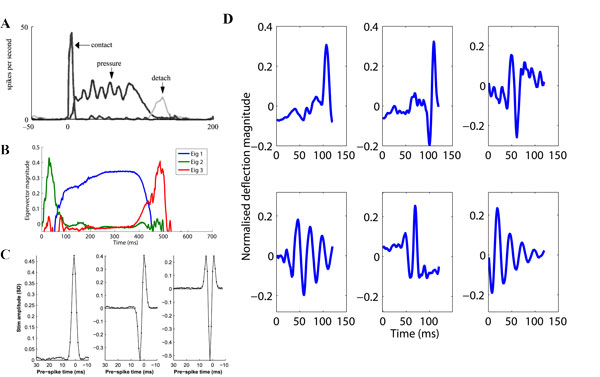
**Neuron and model responses to whisker deflection**. **A **Primary afferent responses. **B **First 3 principal eigenvectors from PCA on artificial data. **C **VPm thalamus responses. **D **Subset of sparse bases

Early sensory encoding appears to follow principles of efficiency and sparsity in both vision and audition [1]. Can a similar sparse coding approach describe the response properties of rodent afferent pathway neurons? The connectivity and general receptive field properties of this system have been characterized, barrel cortex in particular. However the response properties of the afferent pathway neurons have not been described beyond the trigeminal ganglion and VPm neurons of the first lemniscal pathway, partly due to the multi-whisker connectivity of these remaining pathways and the difficulty of stimulating such a system. Primary afferent neurons have been labeled as slowly adapting and rapidly adapting units [2], Figure 1A, and VPm neurons have been described as a population of diverse, precise kinetic feature detectors [3] Figure 1C. Can these responses be explained by sparse coding principles, tuned to object properties in the world? Early work applying a sparse ICA to spectrogram images from artificial whisker data predicted spectro-temporal receptive fields in cortex [4], of which there is tentative evidence [5]. However, rodents are susceptible to velocity-amplitude discrimination 'illusions', suggesting that whisker deflections are not encoded with spectro-temporal precision [5]. We tested a non-linear sparse coding approach [6] on multiple sets of artificial whisker time series data [7,8]. Initial results are shown in Figure 1. The first 3 principle eigenvectors from PCA analysis (Figure 1B) resemble primary afferent response properties (Figure 1A**)**, and some sparsely coded basis functions (Figure 1D), resemble VPm thalamus responses (Figure 1C). We hope to evaluate this approach by verifying model and neural response similarity, and by extension to multi-whisker stimuli.
